# New Model for Predicting the Presence of Coronary Artery Calcification

**DOI:** 10.3390/jcm10030457

**Published:** 2021-01-25

**Authors:** Samel Park, Min Hong, HwaMin Lee, Nam-jun Cho, Eun-Young Lee, Won-Young Lee, Eun-Jung Rhee, Hyo-Wook Gil

**Affiliations:** 1 Department of Internal Medicine, Soonchunhyang University Cheonan Hospital, Cheonan 31151, Korea; samelpark17@schmc.ac.kr (S.P.); chonj@schmc.ac.kr (N.-j.C.); eylee@sch.ac.kr (E.-Y.L.); 2 Department of Software Convergence, Soonchunhyang University, Asan 31538, Korea; mhong@sch.ac.kr (M.H.); leehm@sch.ac.kr (H.L.); 3 Institute of Tissue Regeneration, College of Medicine, Soonchunhyang University, Cheonan 31151, Korea; 4 BK21 FOUR Project, College of Medicine, Soonchunhyang University, Cheonan 31151, Korea; 5 Division of Endocrinology and Metabolism, Department of Internal Medicine, Kangbuk Samsung Hospital, School of Medicine, Sungkyunkwan University, Seoul 03181, Korea; wonyoung2.lee@samsung.com

**Keywords:** coronary artery calcium score, prediction model, vascular calcification

## Abstract

Coronary artery calcification (CAC) is a feature of coronary atherosclerosis and a well-known risk factor for cardiovascular disease (CVD). As the absence of CAC is associated with a lower incidence rate of CVD, measurement of a CAC score is helpful for risk stratification when the risk decision is uncertain. This was a retrospective study with an aim to build a model to predict the presence of CAC (i.e., CAC score = 0 or not) and evaluate the discrimination and calibration power of the model. Our data set was divided into two set (80% for training set and 20% for test set). Ten-fold cross-validation was applied with ten times of interaction in each fold. We built prediction models using logistic regression (LRM), classification and regression tree (CART), conditional inference tree (CIT), and random forest (RF). A total of 3302 patients from two cohorts (Soonchunhyang University Cheonan Hospital and Kangbuk Samsung Health Study) were enrolled. These patients’ ages were between 40 and 75 years. All models showed acceptable accuracies (LRM, 70.71%; CART, 71.32%; CIT, 71.32%; and RF, 71.02%). The decision tree model using CART and CIT showed a reasonable accuracy without complexity. It could be implemented in real-world practice.

## 1. Introduction

Coronary artery calcification (CAC), a feature of coronary atherosclerosis [1], is a well-known risk factor for cardiovascular disease (CVD) [2]. Notably, a CAC score can be implemented for risk stratification when a risk decision is uncertain [2]. Measuring a CAC score may be a beneficial approach, especially for patients with atherosclerotic cardiovascular disease (ASCVD) risk of 5–7.5% [3]. The absence of CAC is associated with a lower incidence rate of CVD [4]. However, measuring a CAC score can be burdensome in terms of the financial aspects of patients and insurance, although previous cost-effectiveness analyses have revealed that CAC testing is cost-effective for asymptomatic patients [4,5,6].

Results of the Multi-Ethnic Study of Atherosclerosis (MESA) have shown that a CAC score is affected by traditional risk factors, including age, male sex, white ethnicity, hypertension (HTN), body mass index (BMI), diabetes mellitus (DM), smoking, and family history of CVD [7,8]. We have shown that insulin resistance [9], lipoprotein(a) [10], hemoglobin glycation index [11], non-alcoholic fatty liver disease (NAFLD) [12], and systemic inflammation [12] are related to progression of CAC in Koreans, similar to the results of the MESA study. Cross-sectionally, we also have shown that metabolic risk factors [13,14,15,16,17], age [18], and non-alcoholic fatty liver disease [19] are associated with incident presence of CAC, defined as a CAC score > 0.

Given the association between traditional risk factors of CVD and CAC mentioned above, we hypothesized that models to predict a patient’s CAC score could be built. For example, we have previously built a model to predict a CAC score > 0 using criteria for metabolic syndrome [16]. Based on the ability of a CAC score to guide risk management of CVD, such a model could contribute to the re-grading of risk prediction from clinical perspectives. Thus, the purpose of this study was to build a model to predict the presence of coronary artery calcification and evaluate the discrimination and calibration power of the model.

## 2. Materials and Methods

### 2.1. Study Population and Design

We merged two cohorts from Soonchunhyang University Cheonan Hospital (Cheonan, Korea) and Kangbuk Samsung Hospital of Sungkyunkwan University (Seoul, Korea). The cohort from Soonchunhyang University Cheonan Hospital was built for our previous study (under review), in which we tried to show that CAC score might be associated with rapid renal deterioration. Briefly, we collected data of patients who underwent cardiac-computed tomography at Soonchunhyang University Cheonan Hospital, Cheonan, Korea, between January 2010 and July 2012. Patients with baseline estimated glomerular filtration rate (eGFR) ≥ 60 mL/min per 1.73 m^2^ and followed up for more than one year were enrolled. 

Another cohort consisted of patients from the Kangbuk Samsung Health Study—a health check-up program at the Health Promotion Center of Kangbuk Samsung Hospital. Detailed methods are described in our previous reports [9,11]. In short, these studies enrolled 2411 patients whose CAC scores were measured between January 2010 and December 2010. They measured the CAC score for the general check-up of the coronary artery, albeit without any symptoms. Patients did not have a history of diabetes, ischemic stroke, or coronary artery disease. They had repeated measurements of CAC score between January 2014 and December 2014. We used follow-up data in 2014 as CAC scores were higher in 2014 than those in 2010. Patients with ages between 40 and 75 years were included because the evidence and guidelines on whether to start statin therapy was robust for this age group [2].

The study protocol was reviewed and approved by the Institutional Review Board at each center (Soonchunhyang University Cheonan Hospital, Cheonan, Korea, SCHCA 2020-10-015; and Sungkyunkwan University Kangbuk Samsung Hospital, Seoul, Korea, KBSMC 2020-12-008). The requirement of informed consent was waived because of its retrospective design. This study was conducted following the principles of the Declaration of Helsinki.

### 2.2. Covariates and STUDY Outcome

Variables available in both cohorts were used, including age, sex, eGFR, prior history of HTN, DM, non-high-density lipoprotein (non-HDL) cholesterol, and BMI. As the status of current smoking was not collected for the Cheonan cohort, we could not assess the effect of smoking on the model. The CAC score was categorized into two groups: CAC score of 0 (negative CAC score) or not (positive CAC score group). 

### 2.3. Statistical Analyses

All statistical analyses were performed using R software version 4.0.2 (The R Foundation for Statistical Computing, Vienna, Austria). Continuous variables are expressed as mean ± SD or median (interquartile ranges), as appropriate. Categorical data are presented as count (percentage). Groups were compared using Student’s *t*-test or the Mann–Whitney test with regard to the distribution of variables for normally distributed continuous variables, as appropriate. For categorical variables, Pearson’s chi-squared test or Fisher’s exact test was used, as appropriate. 

The data set was divided into two groups: 80% for training and 20% for test. The training set was divided into ten folds for cross-validation. Each fold consisted of 90% of training data and 10% of validation data. The first model was built using the training data from the first fold. Next, the model was validated, and its accuracy was calculated using the validation data from the same fold. The second model was built and validated using training and validation data of the second fold, respectively. The process continued until a tenth model was built. This process was repeated ten times. As a result, 100 models were built. Among these models, the model with the highest accuracy was selected. Through these processes, we attempted to ensure the generalizability of the model and prevent overfitting.

Logistic regression, classification and regression tree (CART) [20], conditional inference tree [21], and random forest [22] were used. As covariates, age, sex, BMI, non-HDL cholesterol, eGFR, HTN, and DM were used. When creating logistic models, all continuous variables were log-transformed. Interactions between age and other variables were added to estimate coefficients. After a model construction, another model with the lowest Akaiki information criterion (AIC) was calculated from each fold’s initial model. The regression tree using the CART method was optimized by pruning based on standard errors. Using the CART and conditional inference method, each regression tree model was rebuilt after mutating continuous variables (including age, eGFR, BMI, and non-HDL cholesterol) to categorical type, because categorical variables were more intuitive and could be more easily used. Age was categorized based on decades. Based on a Korean guideline on non-HDL cholesterol management targets according to risk category, non-HDL cholesterol was categorized into <100, 100–129, 130–159, 160–189, and ≥190 [23]. BMI was categorized into <25 or ≥25 (obese) based on the WHO classification for obesity in Asians [24]. eGFR was grouped into ≥90 or 60–89 according to the Kidney Disease: Improving Global Outcomes (KDIGO) guideline [25].

## 3. Results

### 3.1. Characteristics of the Cohorts

**Figure 1:**
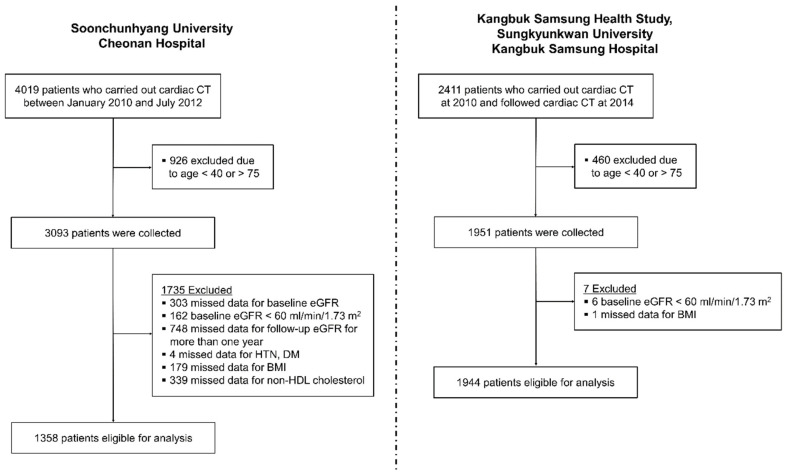
Flow chart showing patient enrollment process. Abbreviations: CT, computed tomography; eGFR, estimated glomerular filtration rate; HTN, hypertension; DM, diabetes mellitus; BMI, body mass index.

Figure 1 depicts the algorithm to enroll patients in both cohorts. The cohort from Soonchunhyang University Cheonan Hospital consisted of 4019 patients. Among them, 2661 patients were excluded as demonstrated in Figure 1. As data of the Cheonan cohort were collected in order to show eGFR trajectories, patients without eGFR data for more than one year were excluded. Finally, a total of 3302 patients (1358 from the Cheonan cohort and 1944 from the Kangbuk Samsung Health Study) remained in the study.

Baseline characteristics of the cohort are demonstrated in Table 1. All patients aged between 40 and 75 years and had eGFR ≥ 60 mL/min per 1.73 m^2^. Among these patients, 1263 (29.2%) had a CAC score above 0 (positive CAC score). In the Cheonan cohort, patients were older (61 (53–69) years vs. 46 (43–49) years) and had a higher percentage of those with a medical history of HTN (59.1% vs. 17.6%) and DM (33.5% vs. 2.1%). They also had higher CAC scores (9 (0–129) vs. 0 (0–1)) than those in cohort of the Kangbuk Samsung Health Study. Because the cohort of the Kangbuk Samsung Health Study consisted of individuals enrolled in health examination programs for workers, it showed a more male predisposition. In addition, patients were younger and less likely to have other comorbidities (i.e., healthier than the Cheonan cohort).

### 3.2. Logistic Regression Models

**Figure 2:**
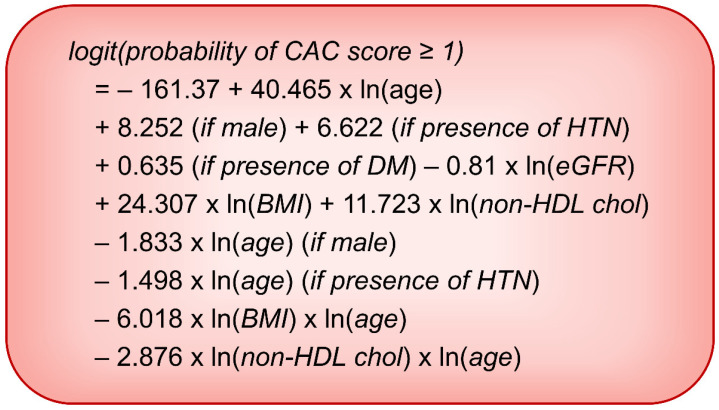
Equation based on the logistic regression model predicting coronary artery calcification score ≥ 1. Abbreviations: CAC, coronary artery calcification; HTN, hypertension; DM, diabetes mellitus; eGFR, estimated glomerular filtration rate; BMI, body mass index; non-HDL chol, non-high density lipoprotein cholesterol.

**Figure 3:**
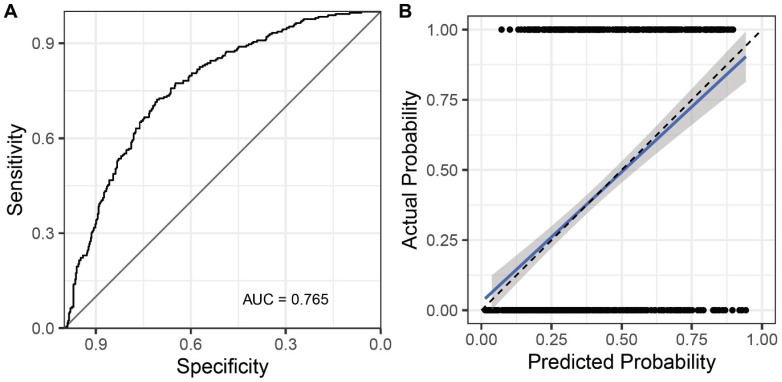
Receiver operator characteristics (ROC) curve (**A**) and calibration plot (**B**) of the logistic regression prediction model. Abbreviation: AUC, area under the ROC curve.

Multivariable logistic regression models are presented in Table 2. The best AIC model was calculated from a basic model that included all variables and interaction rim with log-transformed age. In the best AIC model, interaction rims, including DM * Ln (age) and Ln (eGFR) * Ln (age), were removed from the model. The final logistic regression estimation equation is expressed in Figure 2. Figure 3 shows the receiver operating characteristic (ROC) curve and calibration curve of the final logistic model. The area under the curve (AUC) of the model was 0.765. Accuracy, sensitivity, and specificity of the logistic regression model were 70.71%, 49.60%, and 83.78%, respectively (Table 3).

### 3.3. Classification Trees

**Figure 4:**
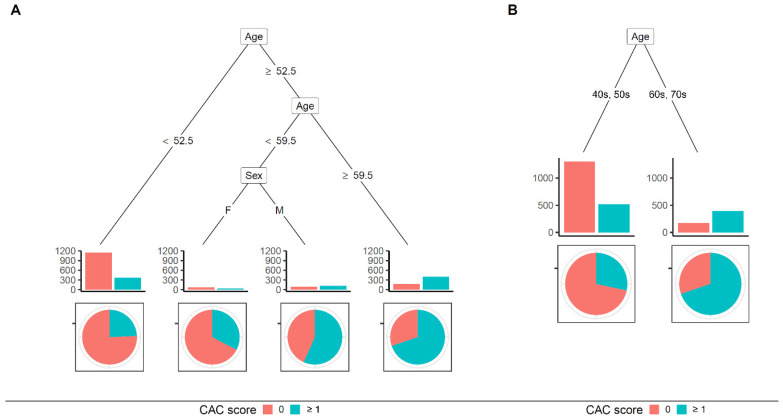
Classification and regression tree (CART) for predicting coronary artery calcium score ≥1. (**A**) CART model using continuous variables; (**B**) CART model using categorically transformed variables. Among various variables, age and sex remained as significant classifiers. Abbreviations: CAC, coronary artery calcification.

Regression trees using the CART method are depicted in Figure 4. In the basic model (using continuous variables), if a patient was above ≥59.5 years in age or was a male with age ≥ 52.5 years, the patient was classified to have a positive CAC score (Figure 4A). As cut-off values were not intuitable, we changed continuous variables to categorical ones, as described above. Based on the new model, if a patient’s age was in the 60s or 70s, the patient was classified into a group with a positive CAC score (Figure 4B). Accuracy, sensitivity, and specificity of the model using continuous variables were 71.32%, 55.16%, and 81.33%, respectively (Table 3). They were 69.35%, 40.08%, and 87.47%, respectively, in the model after categorical transformation (Table 3).

**Figure 5:**
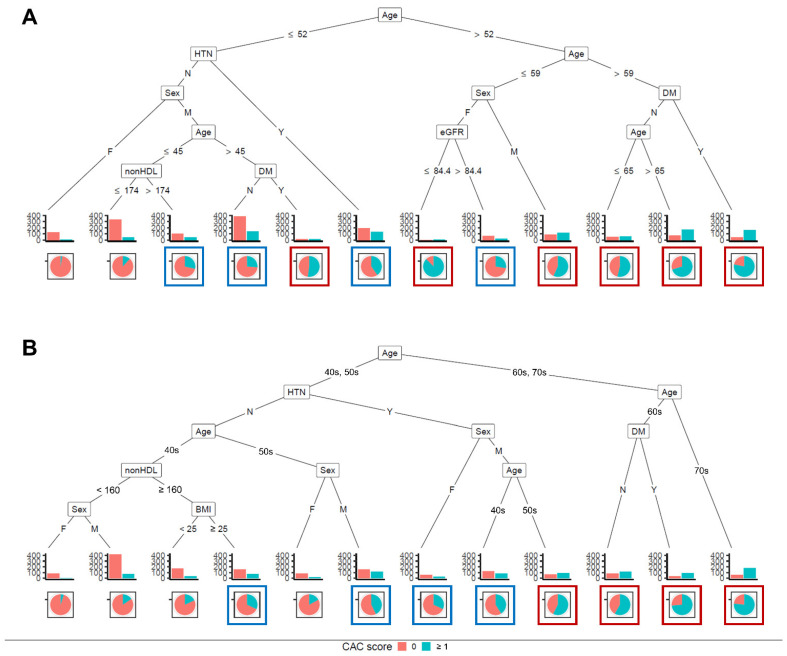
Conditional inference tree for predicting coronary artery calcification score ≥1. (**A**) Conditional inference tree using continuous variables; (**B**) Conditional inference tree using categorically transformed variables. Blue and red boxes denote intermediate (25–49%) and high (≥50%) risks, respectively. Abbreviations: HTN, hypertension; DM, diabetes mellitus; eGFR, estimated glomerular filtration rate; non-HDL chol, non-high density lipoprotein cholesterol; CAC, coronary artery calcification; BMI, body mass index.

In addition, conditional inference trees were trained. These trees were more complex than those using the CART method (Figure 5). Conditional inference trees using continuous variables (Figure 5A) and categorically transformed variables (Figure 5B) were built. In the model with categorical transformation, an age of 60 or 70 was associated with a positive CAC score (10th–12th terminal node in Figure 5B). Male patients aged between 50 and 59 with HTN were associated with a positive CAC score (9th terminal node in Figure 5B). After continuous variables were transformed to categorical ones, accuracy, sensitivity, and specificity decreased similarly to those in the regression tree model using the CART method (Table 3).

**Figure 6:**
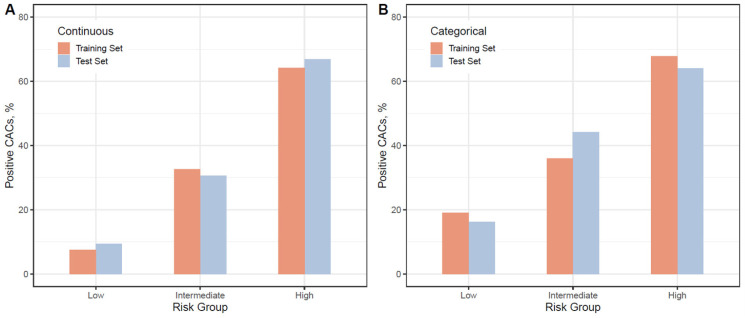
Probability of training set and test set according to risk group estimated using conditional inference trees. (**A**) Conditional inference tree using continuous variables; (**B**) Conditional inference tree using categorically transformed variables. Risk probabilities were categorized into three groups: low (<25%), intermediate (25–49%), and high (≥50%). Positive CAC score represents CAC score ≥ 1. Abbreviation: CACs, coronary artery calcification score.

Next, in conditional inference trees, risk probabilities were categorized into low (<25%, no box), intermediate (25–49%, blue box), and high (≥50%, red box) (Figure 5). There were concordances in risk probabilities between the training set and the test set (Figure 6). In the model using continuous variables, the probability of having a positive CAC score in the low-risk group was less than 10% (Figure 6A). It was lower than those in the model using categorical variables (Figure 6B). We could infer that female patients aged ≤ 52 years without having HTN and non-hypertensive male patients aged ≤ 45 years with non-HDL cholesterol ≤ 174 mg/dL were less likely to have a positive CAC score.

Contrary to expectations, the random forest did not improve the accuracy compared with the logistic regression model (70.71% in the logistic regression model vs. 71.02% in the random forest model, Table 3).

## 4. Discussion

Our study showed that the CAC score could be predicted based on clinical demographics and laboratory data. Recently, the importance of CAC score in the scope of cardiovascular preventive medicine has been rising, especially for guiding when to begin statin therapy. However, taking a CT scan to estimate the CAC score is controversial. Our results might help physicians select patients who need coronary artery calcium CT. The complexity of our logistic model exerted the necessity for a web-based or program-based approach. Therefore, we used a decision tree, a more intuitable option. The CART and the conditional inference tree built in this study showed an acceptable accuracy—about 70% (Table 3). Based on the CART model, coronary artery calcium CT could be needed for patients with an age of 60 years or more or male patients with an age of 53 years or more (Figure 4A). The conditional inference tree showed more complex results. Therefore, we focused on the tree model using categorical variables. If we use a 50% prevalence of positive CAC score as needing a CT scan, the following patients could be recommended for a CT scan: (1) patients with an age in the 60s or 70s and (2) male patients with an age in the 50s and hypertension (Figure 5B).

CAC is the most robust risk factor associated with future adverse events among all risk factors studied so far [26]. The MESA study has shown that CAC scores are associated with a higher risk for incident CHD and that CAC scores have predictive value in addition to standard risk factors [27]. When the CAC score was included, the MESA risk score significantly improved risk prediction (C-statistic from 0.75 to 0.80, *p* < 0.0001) [28]. The predictability of the Framingham Risk Score for CHD was improved when the CAC score was added, especially in patients with a Framingham Risk Score of at least 10% [29]. The incident ASCVD risk of 10-year risk was strongly associated with the CAC score (doubling CAC was estimated to have a 14% increase in ASCVD risk) [30]. 

CAC score is closely associated with a patient’s demographic data, including race, sex, and age, as demonstrated in the MESA study [31]. The cardiovascular risk score as pathological determinants of atherosclerosis in youth (PDAY) could predict future CAC score, suggesting a relationship between CAC score and traditional cardiovascular risks [32]. The PDAY risk score consists of age, sex, non-HDL and HDL cholesterol, smoking, blood pressure, BMI, and glycol-hemoglobin (HbA1c) [32]. DM also contributes to CAC, both in incident CAC and the progression of CAC [7,8]. Given the association between CAC score and traditional cardiovascular risks, including laboratory and demographic data, the prediction of CAC score based on these variables is a reasonably conceivable approach. Therefore, the prediction model of the CAC score could be helpful for clinicians, especially in cost-efficacy, if the accuracy could be precisely advanced and if the model could be validated in the future. Recently, Lee et al. have reported a promising way to predict a CAC score of 100 or more using machine learning algorithms [33]. 

Since the power of a CAC score of 0 is well known, a model is needed to predict whether a CAC score is 0 or not. Alongside the accuracy of the model, it should be readily applicable in real clinical situations. In this study, logistic regression models showed the best C-statistics with moderate accuracies (Table 3). However, a program to auto-calculate risk is necessary because of the complexity of the equation (Figure 2). The regression tree builds simple decision trees using a recursive binary splitting algorithm. It is a simple method to interpret and implement [20,21]. In the present study, conditional inference trees showed a loss in accuracy. However, the extent of loss in accuracy was acceptable, even when variables were transformed into categorical ones (Table 3). 

Our study has several limitations. First, the cohorts used in this study were enrolled for other previous studies and their data were collected retrospectively. This could lead to biases. Second, the cohort from the Cheonan hospital did not have information for smoking. Therefore, we could not integrate smoking status, an important confounding factor, to our model. Third, the prevalence of diabetes in our cohort was too low (only 15%). This could limit our study to general clinical practice because the patient who needs to know a CAC score is likely to be diagnosed with diabetes. Fourth, all participants in our study were Koreans. It is a weakness of our study because the MESA study showed that race could also affect CAC score [31]. Fifth, external validation was not performed. Although we used k-folds cross-validation to minimize risks of overfitting, validation of the prediction model using other data sets is necessary in the field. Lacking validation of models was our limitation.

## 5. Conclusions

Despite the limitations, CAC scores might be predictable based on patients’ demographic and laboratory data. This approach could help clinicians guide personalized therapy in the prevention of ASCVD, although further validation with other studies is warranted. 

## Figures and Tables

**Table 1:** Clinical characteristics of two cohorts enrolled for this study.

Variables	Total Cohorts (*n* = 3302)	Cheonan (*n* = 1358)	Kangbuk Samsung (*n* = 1944)
Age, year	49 (45–58)	61 (53–69)	46 (43–49)
40–49, *n* (%)	1708 (51.7)	196 (14.4)	1512 (77.8)
50–59, *n* (%)	815 (24.7)	399 (29.4)	416 (21.4)
60–69, *n* (%)	453 (13.7)	439 (32.3)	14 (0.7)
70–75, *n* (%)	326 (9.9)	324 (23.9)	2 (0.1)
Male, *n* (%)	2502 (75.8)	718 (52.9)	1784 (91.8)
HTN, *n* (%)	1145 (34.7)	803 (59.1)	342 (17.6)
DM, *n* (%)	495 (15.0)	455 (33.5)	40 (2.1)
eGFR, ml/min per 1.73 m^2^	97.9 (88.3–104.5)	98.4 (90.5–105.1)	97.5 (87.7–104.2)
≥90, *n* (%)	2307 (69.9)	1029 (75.8)	1278 (65.7)
60–89, *n* (%)	995 (30.1)	329 (24.2)	666 (34.3)
BMI, kg/m^2^	24.6 (22.8–26.7)	24.7 (22.7–26.9)	24.6 (22.9–26.6)
<25, *n* (%)	1802 (54.6)	732 (53.9)	1070 (55.0)
≥25, *n* (%)	1500 (45.4)	626 (46.1)	874 (45.0)
non-HDL, mg/dL	146 (120–173)	135 (109–165)	153 (129–177)
<100, *n* (%)	376 (11.4)	246 (18.1)	130 (6.7)
100–129, *n* (%)	727 (22.0)	359 (26.4)	368 (18.9)
130–159, *n* (%)	987 (29.9)	355 (26.1)	632 (32.5)
160–189, *n* (%)	747 (22.6)	240 (17.7)	507 (26.1)
≥190, *n* (%)	465 (14.1)	158 (11.6)	307 (15.8)
CACS, units	0 (0–26)	6 (0–129)	0 (0–1)
0, *n* (%)	2039 (61.8)	601 (44.3)	1438 (74.0)
1–100, *n* (%)	790 (23.9)	375 (27.6)	415 (21.3)
>100, *n* (%)	473 (14.3)	382 (28.1)	91 (4.7)

Abbreviations: HTN, hypertension; DM, diabetes mellitus; eGFR, estimated glomerular filtration rate; BMI, body mass index; non-HDL, non-high density lipoprotein cholesterol; CACS, coronary artery calcification score.

**Table 2:** Results of multivariable logistic regression models.

Variables	Basic Model		Best AIC Model	
	Coefficients, β	*p* Value	Coefficients, β	*p* Value
(Intercept)	−182.013	<0.001	−161.37	<0.001
Ln (Age)	45.716	<0.001	40.465	<0.001
Male	8.516	0.011	8.252	0.013
HTN	6.302	0.011	6.622	0.007
DM	3.917	0.263	0.635	<0.001
Ln (eGFR)	3.259	0.708	−0.81	0.035
Ln (BMI)	23.992	0.008	24.307	0.007
Ln (non-HDL)	12.269	0.003	11.723	0.004
Interaction				
Sex * Ln (age)	−1.898	0.021	−1.833	0.025
HTN * Ln (age)	−1.419	0.023	−1.498	0.015
DM * Ln (age)	−0.81	0.347		
Ln (eGFR) * Ln (age)	−1.035	0.638		
Ln (BMI) * Ln(age)	−5.94	0.009	−6.018	0.008
Ln (non-HDL) * Ln (age)	−3.014	0.003	−2.876	0.005

Note: Akaiki information criterion (AIC): basic model, 2638.4; Best AIC model, 2635.4. Multivariable logistic regression models with full variables and best AIC. Continuous variables were log-transformed. To prevent overfitting, 10-folds cross-validation with 10 times of iteration was done. Among these models, the model with the best AIC was selected. Abbreviations: HTN, hypertension; DM, diabetes mellitus; eGFR, estimated glomerular filtration rate; non-HDL, non-high density lipoprotein cholesterol.

**Table 3:** Performances of models using logistic regression, classification and regression tree, conditional inference tree, and random forest.

	C-Statistics	Kappa	Acc and 95% CI	Sen	Spe	PPV	NPV
LRM	0.765 (0.728–0.801)	0.350	70.71 (67.08–74.16)	49.60	83.78	65.45	72.86
CART	0.690 (0.652–0.728)	0.375	71.32 (67.70~74.75)	55.16	81.33	64.65	74.55
CARTcat	0.638 (0.603–0.672)	0.298	69.35 (65.67–72.85)	40.08	87.47	66.45	70.22
CIT	0.751 (0.714–0.788)	0.379	71.32 (67.70–74.75)	56.75	80.34	64.13	75.00
CITcat	0.759 (0.722–0.796)	0.334	69.95 (66.29–73.43)	48.81	83.05	64.06	72.38
RF	0.753 (0.715–0.791)	0.355	71.02 (67.39–74.46)	49.21	84.52	66.31	72.88

Note: No information rate (NIR), 61.76. All models showed better accuracies than NIR. Abbreviations: LRM, logistic regression model; CART, classification and regression tree; CARTcat, classification and regression tree using categorically transformed variables; CIT, conditional inference tree; CITcat, conditional inference tree using categorically transformed variables; RF, random forest; Kappa, Cohen’s kappa; Acc, accuracy; CI, confidence intervals; Sen, sensitivity; Spe, specificity; PPV, positive predictive value; NPV, negative predictive value.

## Data Availability

Data sharing not applicable.
